# The effect and role of environmental conditions on magnetosome synthesis

**DOI:** 10.3389/fmicb.2014.00049

**Published:** 2014-02-11

**Authors:** Cristina Moisescu, Ioan I. Ardelean, Liane G. Benning

**Affiliations:** ^1^Department of Microbiology, Institute of Biology BucharestBucharest, Romania; ^2^School of Earth and Environment, University of LeedsLeeds, UK

**Keywords:** magnetotactic bacteria, biomineralization, environmental conditions, magnetite characteristics, biogeochemistry

## Abstract

Magnetotactic bacteria (MTB) are considered the model species for the controlled biomineralization of magnetic Fe oxide (magnetite, Fe_3_O_4_) or Fe sulfide (greigite, Fe_3_S_4_) nanocrystals in living organisms. In MTB, magnetic minerals form as membrane-bound, single-magnetic domain crystals known as magnetosomes and the synthesis of magnetosomes by MTB is a highly controlled process at the genetic level. Magnetosome crystals reveal highest purity and highest quality magnetic properties and are therefore increasingly sought after as novel nanoparticulate biomaterials for industrial and medical applications. In addition, “magnetofossils,” have been used as both past terrestrial and potential Martian life biosignature. However, until recently, the general belief was that the morphology of mature magnetite crystals formed by MTB was largely unaffected by environmental conditions. Here we review a series of studies that showed how changes in environmental factors such as temperature, pH, external Fe concentration, external magnetic fields, static or dynamic fluid conditions, and nutrient availability or concentrations can all affect the biomineralization of magnetite magnetosomes in MTB. The resulting variations in magnetic nanocrystals characteristics can have consequence both for their commercial value but also for their use as indicators for ancient life. In this paper we will review the recent findings regarding the influence of variable chemical and physical environmental control factors on the synthesis of magnetosome by MTB, and address the role of MTB in the global biogeochemical cycling of iron.

## Introduction

Biomineralization represents the process by which living organisms produce minerals (Walcott et al., 1979). Biomineralization is a widespread phenomenon, and all six taxonomic domains include members capable of inducing the synthesis of biominerals (Clark and Evans, 1997; Kirschvink et al., 2001). Minerals formed through biological means can be extracellular or intracellular. They are diverse and they often have specific functions. For example, Fe_3_O_4_ biominerals act as magnetic sensors (Hesse, 1994; Lohmann et al., 2001), CaCO_3_ protects against predation (Gower, 2008), CaCO_3_, CaSO_4_, or BaSO_4_ are important as gravitational sensors (Halstead, 1994) while Fe_2_O_3_biominerals passivate surfaces and help reduce corrosion (Rothman and Wieland, 1996).

Prokaryotes play a major role in the deposition and weathering of minerals in the Earth's crust (Frankel and Bazylinski, 2003; Lefèvre and Bazylinski, 2013). Yet, inorganic, metal-rich intracellular minerals are not so commonly found in the prokaryotic genera (Fortin and Langley, 2005). The synthesis of minerals by prokaryotes can be classified into biologically induced mineralization (BIM) and biologically controlled mineralization (BCM) (Lowenstam, 1981; Lowenstam and Weiner, 1989). Minerals that form by BIM processes generally nucleate and grow extracellularly, as an unintended and uncontrolled consequence of metabolic activities. These minerals (also often called biominerals) are however most often characterized by poor crystallinity, broad particle-size distributions, and a lack of specific crystal morphologies. Therefore, BIM is equivalent to inorganic mineral formation under equivalent environmental conditions. Furthermore, the minerals produced by BIM are in general indistinguishable from the minerals produced by purely inorganic chemical reactions (Frankel and Bazylinski, 2003). In contrast, in BCM, minerals are usually deposited on or within the cell, the organism exerting a significant degree of control over the nucleation and growth of the minerals and thus over the composition, size, habit, and intracellular location of the minerals (Bazylinski and Frankel, 2000). The BCM process is therefore a highly controlled process at the metabolic and genetic level. These are true biominerals. As the topic of this paper is the intracellular biomineralization in bacteria, the BIM process will not be further discussed and all our focus will be on BCM biomineralization with specific emphasis on iron biominerals.

One of the most interesting and most studied examples of BCM, with respect to the synthesis of Fe minerals, is the formation of bacterial magnetosomes (Lower and Bazylinski, 2013). Microbial magnetosomes represent a special category of intracellular organelles that are synthesized by magnetotactic bacteria (MTB), which use the magnetosomes for geomagnetic navigation in their aquatic habitat. The unique characteristics and properties of magnetosomes that will be discussed below have in the last few decades attracted huge interest, because their properties can be exploited for a variety of applications. These span many diverse disciplines from microbiology, cellular biology, geobiology, and bio(nano)technology (Bazylinski and Frankel, 2000; Lang et al., 2007; Schuler, 2008). Furthermore, magnetosome biomineralization and their assembly in chains is of great interest for the production of biologically inspired magnetic materials, and they have also been suggested as potential biomarker for detection of extant life signatures on other planets (McKay et al., 1996).

The formation of magnetosomes represents a fascinating example of how apparently simple organisms can translate genetic information into extremely complex inorganic cellular structures (Jogler and Schüler, 2009). A plethora of fundamental biomineralization mechanisms are crucial during magnetosome synthesis and thus MTB can serve as a relatively simple and accessible model for the study of biomineralization processes in general. From the pioneering paper of Blakemore (1975), the subject of magnetosome biomineralization has evolved to a unique and interdisciplinary area of research. It is not the scope of this paper to review the whole literature about the role of MTB and magnetosomes but we will focus our review on the effect of different chemical and physical environmental factors on the synthesis of magnetosome by MTB, and address the role of MTB in the global biogeochemical cycling of iron.

## Magnetosome synthesis

Presently, three minerals with magnetic properties, magnetite (Fe_3_O_4_), maghemite (Fe_2_O_3_), and greigite (Fe_3_S_4_) have been identified in different organisms, from prokaryotes to complex organisms (including humans) (Kirschvink and Hagadorn, 2000). Despite the fact that several studies have addressed the localization and characterization of cellular ultrastructures of ferromagnetic inclusions in various organisms, most of the presently available information comes from the studies of MTB. The intracellular magnetic particles produced by MTB reflect best the optimization processes of natural selection.

### Magnetosome crystals synthesized by MTB

The term “magnetotactic bacteria” represents a morphological, metabolical, and phylogenetical diverse group of prokaryotes capable of passively aligning and actively swimming parallel to the geomagnetic field lines (Bazylinski and Frankel, 2004). MTB were first described in 1891 in the work of Massart (1891) and in the studies of Bellini (1963a,b) as a group of bacteria in which the direction of movement is apparently influenced by the magnetic field. Only their accidental rediscovery in 1974 by Blakemore (1975) re-initiated and stimulated new research activities over the last few decades. MTB represent a collection of diverse bacteria that possess the most unambiguous magnetoreceptive behavior called magnetotaxis (Blakemore, 1982). It was proposed that in natural environments magnetotaxis may enable the cells to locate and maintain an optimal position in the water column or in sediments, and that this aligns with their main metabolic needs (Ardelean et al., 2008). In addition, in contrary to a true taxis, the magnetic assisted taxis could help MTB in their navigation toward optimum growth conditions, when a magnetic field is present, therefore reducing a tri-dimensional search to a more advantageous single-dimensional one (Bazylinski and Frankel, 2004). The great diversity of MTB is expressed in different cell morphologies, a cosmopolitan distribution, and different phylogenetic traits (Fassbinder et al., 1990; Bazylinski and Frankel, 2004; Bazylinski and Lefevre, 2013). Despite their diversity, all MTB are Gram-negative, motile by means of monotrichous, bipolar or lophotrichous flagella, and all possess a microaerophilic (Schleifer et al., 1991; Spring et al., 1993) or anaerobic, sulfate-reducing respiratory metabolism (Bazylinski et al., 1988; Sakaguchi et al., 1993). Furthermore, in MTB cell division occurs at a central point of the cells and the magnetosome chains are cleaved into two even chains with the resulting number of magnetosomes being directly proportional to the cell length (Staniland et al., 2010). The most important characteristic that sets them apart from other bacteria is their ability to synthesize nanometer-sized crystals of a magnetic mineral that is either magnetite (Fe_3_O_4_) (Frankel et al., 1979), greigite (Fe_3_S_4_) (Mann et al., 1990) or both (Bazylinski et al., 1993b, 1995; Lins et al., 2007). The formation of such magnetosomes through a biomineralization process is highly genetically controlled and this leads to magnetic crystals that are of exceptional high purity, specific sizes and shapes and that assemble in well-ordered chains, that ultimately function as an extremely efficient magnetic sensor (Rodgers et al., 1990; Schuler, 1999). Even though the composition, size and morphology of the magnetic crystals may vary from species to species they are highly conserved within the same bacterial species or genus (Bazylinski et al., 1994). The high chemical purity of MTB magnetosomes, and magnetite's in particular (Bazylinski, 1995; Bazylinski and Frankel, 2000), is not surprising. As compared to their mineralogical equivalent, the incorporation of trace elements reduces the magnetic moment of the abiotically formed magnetite particles (Kopp and Kirschvink, 2008). Although for many years it was believed that in magnetite, Fe cannot be replaced by other transition metal ions such as Ti, Cr, Co, Cu, Ni, Hg or Pb, recent studies showed that this is not universally applicable. For example, Ti was discovered in magnetite particles of an uncultured magnetotactic coccus (Towe and Moench, 1981), Mn in the magnetite particles of a brackish-to-marine coccus (Keim et al., 2009), and Co in the magnetite magnetosomes from three species of *Magnetospirillum* (Staniland et al., 2008). The effects that these “impurities” have on magnetosome characteristics are discussed in section The Effect of Chemical Impurities. The second most common mineral type forming MTB magnetosomes is greigite. Compared to magnetite, greigite magnetosome crystals are less strictly controlled, both chemically and crystallographically and some greigite-producers can incorporate up to 10 atom% Cu into their crystals (Bazylinski et al., 1993a; Pósfai et al., 1998). Although the reason for these differences is not yet fully understood, the main difference is most likely linked to the mechanisms of formation of magnetite vs. greigite. In abiotic systems, experimental studies have shown that magnetic nanocrystals of greigite form in reducing environments most often via a nanocrystaline, non-stoichiometric Fe^2+^ precursor, mackinawite (Cahill et al., 2000; Hunger and Benning, 2007; Csákberényi-Malasics et al., 2012), while magnetite forms often via reductive dissolution of an Fe^3+^ precursor, ferrihydrite (Lovley, 1991; Hansel et al., 2003).

The physical volume and shape of individual magnetite crystals determine how well grains function as discrete bar magnets. Both mature magnetite and greigite magnetosome crystals vary in size between 30 and 120 nm (Bazylinski et al., 1994; Frankel et al., 1998; Moisescu et al., 2008; Pósfai et al., 2013). This is also the size range that characterizes the single magnetic domain crystals (SD) (Butler and Banerjee, 1975; Moskowitz, 1995). Although the boundary between the single magnetic and multi magnetic domain for greigite may be at much larger sizes (Hoffmann, 1992), in general, for both minerals, the isolates with sizes smaller than 30 nm fall within the superparamagnetic region (SPM) and the ones greater than 120 nm have multiple magnetic domains and are called multi-domain magnetic crystals (MD). The crystals best suited for magnetoreception are SD crystals, and these are usually preferred and propagated during their evolution into magnetosomes inside MTB.

Biominerals formed by MTB consist of highly uniform crystals with narrow crystal size distributions (CSD) and shape factors (SFD) (Devouard et al., 1998; Arato et al., 2005; Jandacka et al., 2013). In the last few decades various studies (Meldrum et al., 1993; Pósfai and Arato, 2000) have shown that crystal size distribution curves of MTB magnetosomes are normal asymmetric and negatively skewed with sharp cut-offs toward larger sizes. In contrast, magnetite crystals produced in abiotic reactions lead to magnetite crystals with low crystallinity and log normal broad size distributions. The strict control of biomineralization exerted by MTB stops the magnetosomes from growing once they reached a certain, strain-specific size. Only few exceptions to this universal rule are known and these are MTB that produce Gaussian size distributions (Devouard et al., 1998; Pósfai et al., 2001; Arato et al., 2005).

The three most common MTB-produced magnetosome magnetite crystal morphologies are equidimensional cubo-octahedra, elongate hexa-octa hedral prisms, and irregular and elongate tooth, bullet (Mann et al., 1987; Thornhill et al., 1994), or arrowhead (Bazylinski et al., 1995) shapes. Common magnetosome greigite morphologies include equidimensional cubo-octahedra and pleiomorphic elongate rectangular prisms (Bazylinski et al., 1994; Pósfai et al., 1998). Furthermore, under non-stressed environmental conditions the shapes of specific magnetosome crystals appear to be constant within given species or strains for both magnetite and greigite magnetosomes although minor variations of shape and size can occur primarily in greigite magnetosomes (Pósfai et al., 1998). However, as we will discuss below in section Influence of Environmental Factors on Magnetosome Characteristics, stressed environments can induce dramatic changes in shapes and sizes of magnetite magnetosomes.

Idealized crystal habits for magnetite, derived from high-resolution electron microscopic studies, are most often combinations of {100} (cube), {110} (dodecahedron), and {111} (octahedron) forms. The idealized habits of cuboidal magnetosome crystals are cubo-octahedra, composed of {100} + {111} forms with equal development of the six symmetry-related faces of the {100} form and the eight symmetry-related faces of the {111} form (Mann et al., 1984). In MTB with elongate magnetite particles, the crystals are typically elongated along a [111] axis (the “easy” direction of magnetization), with some exceptions (Mann et al., 1987; Taylor and Barry, 2004). Greigite crystals are often elongated along the [100] axis (Pósfai et al., 1998), considered by some the greigite magnetocrystalline “easy” axis (Bazylinski and Moskowitz, 1997).

Crystallographic defects are rare in magnetite magnetosomes grown by MTB under optimal conditions (Devouard et al., 1998), the only deviation from an ideal structure being the spinel-law twins, stacking-fault defects or sub-grain boundaries. With the exception of twinning along the [111] easy axis, crystallographic defects usually reduce the magnetic moment of magnetite particles. In contrast, greigite magnetosome crystals commonly exhibit planar defects along (222) planes, uneven contrast or wrinkles, believed to be associated with the conversion of the precursor mackinawite into greigite (Pósfai et al., 1998; Hunger and Benning, 2007). These differences imply once again that greigite formation by MTB is less well regulated compared to magnetite magnetosome formation and the smaller differences between magnetosome greigite crystals and abiotic greigite crystals could hamper identification of greigite magnetofossils.

### Magnetosome crystals synthesized by other organisms

In the last few decades it was discovered that not only MTB are capable of producing magnetic intracellular inclusions. Other organisms, starting with single cell prokaryotes and even multicellular eukaryotes, may contain various metal or metalloid inclusions with magnetic properties. These are known as non-crystal magnetosomes or magnet-sensitive inclusions (Vainshtein et al., 1997; Kirschvink et al., 2001; Ariskina, 2003; Langley, 2006). These metal-rich magnetic intracellular inclusions seem to have a widespread distribution (in 4 of 6 Kingdoms), similar species specific shapes and sizes, and the producing organisms seem to be apart on the evolutionary scale. However, few details are known about these other organisms that produce such magnetites and additional in depth studies to address their occurrence in prokaryotic genera and in other animals through thorough microbiological, chemical, crystallographic, and high-resolution electron microscopic and spectroscopic analyses are needed. The formation or characteristics of these non-crystal magnetosomes will not be pursued further in the present paper and below we will concentrate only on crystalline magnetosomes found in MTB, disregarding the non-crystal magnetic inclusions found in other species (Ariskina, 2003; Fortin and Langley, 2005; Pósfai and Dunin-Borkowski, 2009).

## Influence of environmental factors on magnetosome characteristics

Despite their relatively ubiquitous distribution, MTB were for a long time considered to be mesophilic and neutrophilic microorganisms with regard to their growth temperature and pH. Only recently, due to the discovery of new thermophilic and alkaliphilic species, have MTB been re-classified as extremophilic (Bazylinski and Lefevre, 2013). These newly discovered extremophilic MTB behave similarly to mesophilic MTB, in that their magnetosomes, although synthesized under extreme conditions, are very similar to magnetosomes formed in moderate environments. This new discovery extends significantly the range of environmental parameters at which MTB can survive, grow, and synthesize magnetosomes. These extremophilic MTB most likely either evolved from other thermophilic or alkaliphilic non-magnetotactic strains, that for some reason, gained the ability to synthesize magnetosomes, or simply from mesophilic MTB that adapted with time to a specific extreme environment, yet without losing the ability to synthesize magnetosomes. However, what happens with MTB when the environmental conditions change rapidly and the bacteria need to adapt fast or do not have time to adapt? How do such abrupt changes affect magnetosome synthesis or bacterial growth? Faivre et al. (2008) suggested that the variation in environmental parameters such as Fe bioavailability, pH and temperature, could have a considerable impact on cell physiology and consecutively on the physical and microstructural characteristics of bacterially formed magnetite crystals. Yet, only few studies have addressed this subject. Below we review all studies that tested the effects and roles of multiple environmental parameter variations on magnetite magnetosome synthesis and discuss how the changes in these parameters can result in changes in the possible commercial value of MTB magnetosome crystal or their potential use as indicators for ancient life.

### The effect of Fe concentration, pH, and temperature

Iron is particularly important in MTB not only for its function as a protein cofactor but mostly for the BCM of the nanometer-sized Fe mineral crystals within their cells. Faivre et al. (2008) showed that the sizes and morphologies of mature magnetosomes synthesized by *M. gryphiswaldense* MSR-1 (DSM 6361) are influenced by the initial Fe availability. Although the mean particle size of the studied magnetosomes was similar to that of the reference magnetosomes, the physical properties such as CSD, aspect ratio, and morphology were significantly different. They showed that an initial Fe starvation period followed by the addition of Fe^3+^ lead to an induced synthesis of magnetosomes with positively skewed broad shape CSDs and irregular morphologies. The majority of the formed crystals revealed a cube-like shape, with small (110) and (111) faces. In these induced cells, a rate of Fe^3+^ uptake of 30 nmol min^−1^ (mg dry weight)^−1^ was measured. This is 30 times higher compared to ~1 nmol min^−1^ (mg dry weight)^−1^ measured for permanently iron-supplemented cells. In addition, the change from a generally cubo-octahedral morphology in the reference magnetosomes, to shapes with nearly equal (100) and (111) faces and little to no expression of (110) faces, indicates that the biological control over magnetite biomineralization by this strain of MTB was highly affected by the variation of environmental parameters. This shows that—at least in this case, Fe uptake rates occupied a key role in controlling magnetosome crystal formation. The authors concluded that the cells could not cope with such high Fe uptake rates, and thus they exerted a lesser biochemical control over the mineralization process. They also concluded that a slower uptake rate will result in the formation of crystals with classical crystal shape and sizes. Furthermore, the Faivre et al. (2008) study also showed that variations in iron uptake need to be taken into consideration when defining and evaluating any morphological biomarker signatures because under varying environmental conditions MTB can form magnetosomes with unexpected morphologies (Faivre et al., 2008).

**Figure 1:**
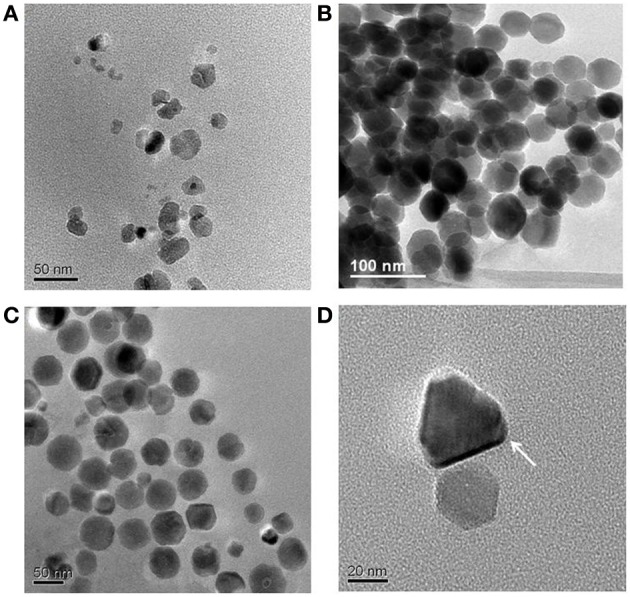
**TEM images of magnetite crystals synthesized by *M. gryphiswaldense* cells grown at 28°C and at different pH values: (A) 6.0; (B) 7.0; (C) 8.0; (D) 9.0**.

In view of this conclusion, our recent study (Moisescu et al., 2011) aimed to mimic other possible environmental variations, such as pH and temperature and to co-evaluate the Fe uptake rates under these changing conditions. We grew first *M. gryphiswaldense* MSR-1 (DSM 6361) at optimum conditions (i.e., pH 7.0 and 28°C) and our Fe uptake rates, crystal morphologies, and sizes were in good agreement with many other MTB studies at optimal conditions (Schuler and Baeuerlein, 1996; Schubbe et al., 2003; Faivre et al., 2008). From these experiments, we determined an Fe uptake rate of *v*_max_ of 0.8 nmol min^−1^ (mg dry weight)^−1^ which is close to most literature values reported for studies with the same strain also grown at optimum conditions. Not surprisingly, when the temperature and especially the pH were shifted away from the optimum (i.e., we varied temperature between 4 and 35°C and pH between 5.0 and 9.0), the ability of MSR-1 to control the biomineralization process was altered. These variations in environmental growth conditions resulted in the synthesis of magnetites with a dramatically changed range in crystal sizes and crystal morphologies (Figures 1A–C) and culminated in the formation of unique pyramidal morphologies that had never been described before in any other biotic or abiotic systems (Figure 1D).

Our results revealed that at ambient conditions when we changed the pH of the growth medium, we observed at all pH values (except at pH 5.0) still high levels of Fe uptake, but interestingly we also observed changed in the formed magnetosomes. Overall, the determined uptake rates were 4–17 times slower than those measured at the optimum pH 7.0. According to Faivre et al. (2008), a slower Fe uptake rate will determine the synthesis of magnetosomes with better-defined sizes and shapes. Apparently this does not apply in the case of pH variations. The magnetites synthesized at pH 6.0 (Figure 1A) were equivalent to immature, poorly developed, or extracellular crystals formed through a BIM process (Devouard et al., 1998; Faivre and Zuddas, 2006; Faivre et al., 2008). These crystals were usually very small and characterized by poor crystallinity, no specific morphology, and the majority fell into the SPM. They had broad, asymmetric, and positively skewed normal CSDs, with sharp cut-offs toward smaller sizes and a SFD < 0.75, characteristics typical for immature or abiotic magnetites. At pH 8.0 (Figure 1C), we found larger SD crystals, with roundish morphologies, and asymmetric negatively skewed CSD and SFD, characteristics more similar to typical intracellular magnetites synthesized by MTB. The main difference observed between crystals formed at pH 6.0 and those formed at pH 9.0 was the well-defined crystal morphology and the appreciably bigger sizes of the pH 9.0 crystals and the fact that they mostly plotted in the SD region. Interestingly, at pH 9.0 an unexpected anisotropic, pyramidal crystal habit was observed (Figure 1D), which makes these crystals unique among both biotic and abiotic magnetites and in principle, this morphology could constitute a physical signature of a biological origin.

The second variable we tested was temperature (Moisescu et al., 2011). In contrast to the changes we observed due to variations in pH, our results revealed that temperature variations had a far weaker effect. Changing temperature during magnetosome growth lead either to a total inhibition of magnetosome synthesis (at 4 and 35°C) or showed no modifications in sizes and shapes of the synthesized magnetites (at 10 and 20°C) compared to the optimal temperature conditions (28°C).

These results suggest that it is not only the Fe uptake rate that influenced the size and morphology of magnetite crystals formed by MTB but that other environmental factors, especially pH, also have a great impact on the number, size, and morphology of magnetite crystals synthesized by MTB.

### The effect of chemical impurities

Many studies showed that MTB cells have a high affinity and specificity for Fe and are capable of up-taking Fe even from very limited concentrations (Nakamura et al., 1993; Schuler and Baeuerlein, 1996; Dubbels et al., 2004). Whereas greigite magnetosomes can contain different metal impurities (Bazylinski et al., 1993a; Pósfai et al., 1998), magnetite was considered for a long time to be of exceptionally high chemical purity, a characteristic that has for a long time been used to discriminate magnetosome magnetite crystals from abiotic crystals.

Recently, however Staniland et al. (2008) showed that magnetosomes containing Co could be produced by three strains of *Magnetospirillum*. *In vivo* Co doping of magnetosomes was achieved (reaching 0.2–1.4% Co) and the magnetite magnetosomes synthesized in the Co systems were slightly larger, more uniform in size and with a narrower CSD, and nearly isotropic or of slightly elongated shapes that aligned parallel to the direction of the chain. An increase in the coercive field of these particles by 36–45% was also attributed to the effect of Co doping. The Co was found to be localized more in the surface layer of the crystal than in the core, indicating that in the initial phases of magnetosome formation, Fe was the preferred magnetosome seed material. Although some crystal imperfections appeared in the Co-doped magnetosomes, so far additional details about these effects are lacking.

Another study followed the Mn accumulation by an uncultured MTB from a coastal lagoon in Rio de Janeiro (Keim et al., 2009). The authors showed that when exposed to a high metal concentration this uncultured strain could incorporate into some of the growing magnetite crystals up to 2.8 atom% Mn with respect to the total metal content (Fe/Mn). In those magnetosomes where high Mn contents were observed, the Mn ions were always colocalized with Fe, and the Mn was homogeneously dispersed throughout the entire magnetite structure and not only on the surface of the crystal as in the case of Co.

These two reports of Co and Mn ions incorporated in magnetite crystals formed by both cultured and uncultured MTB, suggest the possibility that other metal impurities commonly found in inorganic magnetite, such as Ti, Cr, and Zn (Clark and Evans, 1997) could also be incorporated in bacterial magnetite. However, so far, studies done to elucidate the exact mechanisms and effects of such metal impurities are still inconclusive (Towe and Moench, 1981; Keim et al., 2001). This means that the high chemical purity that used to be a prime biosignature of magnetotactic magnetite crystals can no longer be taken as a strict characteristic for the biogenic origin of magnetite crystals.

### The effect of oxygen on magnetite formation under static or dynamic fluid conditions

The majority of MTB species have a microaerophilic or anaerobic respiratory metabolism. Many studies showed that the concentration of O_2_ during MTB growth has a huge influence on magnetite magnetosome synthesis because it can impair the development of magnetosomes (Schuler and Baeuerlein, 1998; Heyen and Schuler, 2003). Therefore, magnetosome development is strictly correlated with a narrow range of (low) O_2_ concentrations.

Popa et al. (2009) evaluated the effects of various levels of initial O_2_ and of various liquid: gas ratios on cultures of *M*. *magneticum* (strain AMB-1). They monitored magnetite magnetosome formation at different initial O_2_ levels, with or without stirring. Their results revealed that under O_2_-stress but stirred (≥45 μM O_2_/150 rpm) 95% of the formed magnetite crystals were dwarf magnetites. These dwarf magnetites consisted of non-euhedral spheroids (~25 nm, with some as small as 10 nm) very similar in shape and size to immature crystals that had formed in equivalent cell cultures grown at optimum O_2_ concentrations. Some of the formed particles were also elongated yet still non-euhedral in shape. In addition, a slightly elevated number of non-aligned magnetite particles per cell (11 ± 2 vs. ~5 ± 2% in cells with normal magnetite particles) were observed. All magnetite crystals that formed under O_2_-stress were SD magnetite, and none showed a SP behavior. The magnetic signatures, Fe^2+^/Fe^3+^, ratios and XRD patterns were comparable to mature magnetite crystals formed in cultures grown at normal O_2_ concentrations (≤18.7 μM O_2_/150 rpm) (Popa et al., 2009). In stirred growth experiments carried out at higher levels of O_2_ (50–100 μM/150 rpm), magnetite biomineralization was strongly inhibited. Conversely, in fully anaerobic conditions (i.e., 0% O_2_) only normal (euhedral, SD) mature crystals formed. These results suggest that O_2_ concentration is in fact important for magnetite synthesis, whereas stirring alone has no other influence than to enhance the O_2_ effect.

These observations are supported by the results of Li and Pan (2012) who showed that under aerobic and anaerobic but using four different stirring growth conditions (anaerobic static, aerobic static, aerobic-80 rpm rotating, and aerobic-120 rpm rotating), the O_2_ parameter was the one parameter that truly affected the biomineralization of magnetite magnetosomes in *M. magneticum* strain AMB-1. When stirred (120 rpm) anaerobic and aerobic conditions were compared, the data revealed that in the presence of high O_2_ contents the cells produced fewer magnetosomes and these were of decreasing grain sizes. The CSD of the magnetosomes was negatively skewed and relatively wide. For the anaerobic and aerobic static cultures predominantly SD particles were observed, while a near normal and narrow distribution with a mixture of SD and SPM particles were observed in the aerobic-80 and −120 rpm stirred cultures. The SFDs indicated a change in crystal shape from more elongated to cubic-like crystals. The magnetosomes produced under the four different stirred conditions were mostly arranged into a single linear fragmented chain. Among the magnetosomes in each chain the frequency of twinned crystals (occasionally even multiple twins) increased gradually, reaching a maximum in the aerobic-120 rpm stirred cultures. High resolution electron microscopy studies confirmed that the magnetosomes formed by these AMB-1 cells were pure magnetite, with a truncated octahedron crystal habit (Li and Pan, 2012). Although the magnetite produced under anaerobic and aerobic stirred growth conditions had similar CSD and SFD, there was a 2 K decrease in the Verwey transition and a 27% decrease in the magnetic coercivity for the aerobically grown magnetites. These changes clearly demonstrate that O_2_ concentration dramatically influences the biomineralization of magnetite magnetosomes confirming the Popa et al. (2009) results. However, the Li and Pan (2012) results also revealed that stirring does not seem to significantly affect magnetosome formation.

### The effect of external magnetic fields on magnetite magnetosome formation

In order to evaluate the role of the geomagnetic field on magnetite magnetosome biomineralization Wang et al. (2008) conducted a set of experiments were a hypomagnetic field (i.e., a weak static magnetic field) less than 500 nT was applied during the culturing of *M. magneticum* strain AMB-1. The results showed that exposure to hypomagnetic field had no significant effects on cell shape but the growth of AMB-1 was restrained during the stationary-phase. Furthermore, exposure to such an external magnetic field during growth increased the percentage of bacteria that contained mature SD magnetosomes in their exponential growth phase. The formed magnetic particles were enlarged although, the amount of Fe depletion from the culture media by cells grown in a 500 nT hypomagnetic field, showed no significant difference from cultures grown in the geomagnetic field. There was also no significant difference in the average particle number per cell between the two groups, but the average size of magnetic particles in cells exposed to hypomagnetic field was larger (>50 nm) and they contained a larger proportion (57%) of SD particles compared to those grown in the geomagnetic field only. These authors also reported that the CSD of the magnetic particles grown only under geomagnetic field had a log-normal distribution with a cut-off toward larger sizes. Conversely, the CSD for the hypomagnetic field group was close to normal distribution. Finally, no significant differences were observed between the SFDs of both groups, and the majority of magnetic particles formed were cubo-octahedrons.

Wang et al. (2008) also showed that using non-magnetic cells (i.e., cells lacking magnetosomes) leads to similar results like in those from the magnetic pre-cultures: enlarged magnetic particles, same Fe depletion and same average number of magnetic particles per cell.

When external fields higher than the Earth's magnetic field were applied (i.e., 0.2 T; Wang and Liang, 2009) to either magnetic or non-magnetic cells of the *M. magneticum* strain AMB-1, these author's results showed that such a static magnetic field could restrain the cellular growth but increase the percentage of cells containing mature magnetosomes by 29%. Although no significant effect was observed on cell shape, an increased number of magnetic particles per cell were observed. In addition, the Fe depletion ability of cells grown under a static high magnetic field was slightly higher (~5%) compared to only geomagnetic field exposed cells. All magnetic crystals that AMB-1 formed when grown in a normal geomagnetic field were closely aligned in chains, whereas an abnormal arrangement was observed in cells exposed to an enhanced static magnetic field (i.e., changes in chain linearity, arrangement of neighboring magnetosomes, and increased number of magnetosome crystals by ~29%).

These results suggest that exposure to magnetic fields, regardless if solely geomagnetic or to an external field play an important role in the biomineralization of magnetosomes. Variations in field strengths seem clearly to affect the control ability of MTB to biomineralize, with consequences mainly with respect to magnetic particles sizes (Wang et al., 2008; Wang and Liang, 2009).

### The effect of nutrient concentrations

Basic nutrients for all cell types are carbon (C), hydrogen (H), oxygen (O), and nitrogen (N). Recently, it was suggested that during MTB growth, iron (Fe) also serves as a nutrient rather than just a storage mineral (Naresh et al., 2012). This was concluded from the fact that Fe was more correlated with C and N, compared to the correlation between C and N which are used mainly as an energy source (C), and for biosynthetic processes (N). When C is limited, cell growth is slower and magnetosome synthesis is slower in contrast to when C is not limited, and magnetosomes are synthesized much faster. In addition, as we discussed above, both Fe and O_2_ concentrations are two of the fundamental controls on magnetosome production in MTB. As to the Fe and N relationship, during the lag and exponential growth phases there is a substantial demand of N to form proteins for assembly of magnetosome vesicles, and to transport and incorporate Fe for magnetosome synthesis. After the vesicles assemble, proteins promote the nucleation of Fe crystals leading to MTB magnetosomes (Naresh et al., 2012). This was clearly demonstrated recently by Siponen et al. (2013), who showed that the magnetosome associated protein MamP plays the crucial role in magnetite crystal growth inside MTB (Siponen et al., 2013).

Interestingly, iron and carbon cycling via magnetosomes has been suggested by Kirschvink and Chang (1984) as being important also in the fossil record. They were the first to propose that magnetosomes preserved as magnetofossils might serve as paleoenvironmental indicators (i.e., paleoxygen or paleocarbon indicators) (see also below) (Kirschvink and Chang, 1984). Hesse (1994) inferred that O_2_ depletion coupled with an increase in organic C flux may have disfavored MTB growth and thus led to the production of elongated crystals at lower O_2_ levels compared to equidimensional particles produced at optimal conditions. Hesse (1994) also found that the abundance of SD magnetite and the ratio of equidimensional to elongate magnetofossils decreased during glacial stages. This led him to hypothesize that these morphological changes may reflect differences in growth rather than in preservation. Likewise, Yamazaki and Kawahata (1998) found that the ratio of equidimensional to elongate magnetofossils was higher in areas where the organic C flux was lower and that a clear link existed between organic C and magnetofossil morphology (Yamazaki and Kawahata, 1998). On the contrary, during glacial intervals an increased organic C content leads to an increase in equidimensional magnetofossils coupled with a decrease in total magnetofossils abundance (Lean and McCave, 1998). Finally, in lake sediments Snowball et al. (1999) revealed a direct relationship between magnetosome concentration and the percentage of total organic C and thus also suggested that the production of magnetosomes by MTB is controlled primarily by the supply of organic C (Snowball et al., 1999). Other chemical parameters have also been inferred to play an important role during the synthesis of MTB magnetites. For example, salinity or nitrate are believed to both strongly influence the number and composition of MTB communities (Simmons et al., 2004; Jogler et al., 2010; Lin and Pan, 2010) yet further studies to better quantify these effects are needed.

Many of these studies show that under certain conditions, magnetosome size, synthesis pathways, etc. can all be affected by environmental factors. Although magnetosome biomineralization has been shown to be a highly controlled BCM process, these studies showed that the role of MTB in providing a suitable chemical environment for intracellular magnetite precipitation is limited when unfavorable environmental conditions are present. In order to fully elucidate the variability in magnetosome synthesis more high-resolution records of these environmental variations must be evaluated in order to provide a full picture of MTB magnetosome formation conditions.

**Figure 2:**
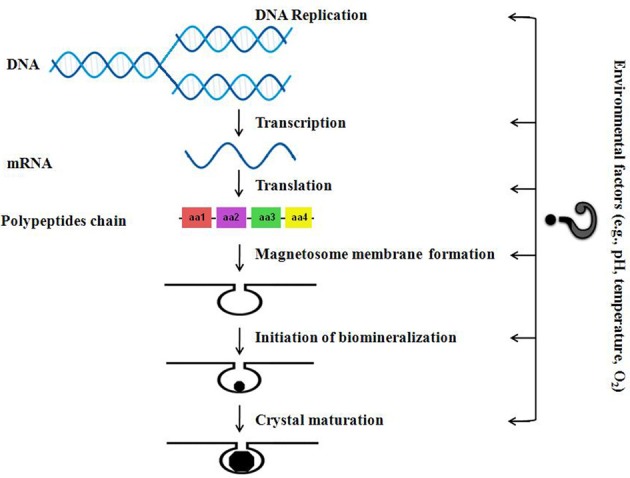
**Schematic diagram about how different environmental factors could possibly affect magnetosome synthesis in MTB (adapted after Murat et al., 2010)**.

Taking into account the proposed main stages of magnetosome formation as described by Murat et al. (2010) and Komeili (2012), we have drawn up a simple schematic diagram (Figure 2) that shows the levels at which environmental factors will most likely affect magnetosome synthesis. It has to be noted however that the vast majority—if not all—of the significant work done on the (bio)chemical reactions leading to magnetosome formation in MTB and any genetic and environmental controls affecting growth, have been derived from studies on pure strains cultivated in the laboratory in organic-rich, well stirred, nutrient-rich media—i.e., most often under optimal conditions. In our opinion, many more laboratory experiments done on pure cultures or microcosms as well as studies of microbiota in natural environments have to be carried out in order to significantly change our understanding of the impact of the plethora of possible environmental factors on the biology of MTB (e.g., magnetosome synthesis, distribution and motility of MTB, competition with other bacteria for resources, etc.). Such studies need to include the quantification of the selective advantages of MTB with magnetosomes (synthesized in different conditions, thus having different shapes, sizes and magnetic properties) over MTB without magnetosomes, and over other heterotrophic microorganisms (belonging to endogenous microbiota). So far, to the best of our knowledge only one such experiment was carried out under laboratory conditions (Smith et al., 2006). These authors tested a pure strain and they took into account only one main parameter: the concentration of oxygen. They argued that, according to their experimental results and the derived theoretical model, the key benefit of magnetotaxis is an enhancement of bacterium's ability to detect oxygen, not an increase in its average speed moving away from high oxygen concentrations (Smith et al., 2006).

## Biomarkers for ancient life

As mentioned above, Kirschvink and Chang (1984) proposed that magnetosomes preserved as magnetofossils could serve as biomarkers for paleoenvironmental conditions. In principle, magnetofossils found in the terrestrial geologic record that possess all or some of the above characteristics in terms of SD, size, shapes, relative purity, etc. are presumed to originate from MTB. This is particularly because inorganically magnetites form via different pathways and lead to different magnetite shapes compared to MTB magnetites. Moreover, because other animals produce only minor amounts of magnetic nanocrystals, and animals are less abundant than bacteria, the straightforward conclusion was that magnetofossils come from MTB. Furthermore, it has been suggested that microorganisms similar to Earth's MTB may have led to similar magnetic crystal signatures on other planets in our solar system (Thomas-Keprta et al., 2000). On Earth, the natural selection of bacterial magnetosomes has left a clear fingerprint on their size, shape, crystallinity, and chemistry, a set of distinctive features that cannot be mimicked yet through any known inorganic processes and this makes magnetofossils relatively easy to identify in the geologic sections.

The development of sensitive techniques to characterize the magnetofossils and infer their origin is the key to a correct identification of MTB in the fossil records. There have been many attempts to establish a coherent set of criteria for the determination of magnetofossils biogenicity (Petersen et al., 1986; Thomas-Keprta et al., 2000, 2001; Clemett et al., 2002; Weiss et al., 2004; Arato et al., 2005; Faivre and Zuddas, 2006; Kopp et al., 2006). In this context, Kopp and Kirschvink (2008) proposed six criteria that can be used when rating possible magnetofossils. These authors also tested these criteria on various geological examples. For their comparison Kopp and Kirschvink (2008) used samples derived from well understood stratigraphic, geochemical, and paleomagnetic contexts, and/or from localities with robust paleomagnetic data (Chang, 1988; Hounslow and Maher, 1996; Montgomery et al., 1998; Maher et al., 1999). Their results showed that such a method and these criteria work very well in distinguishing and maybe even confirming MTB-derived magnetofossils in ancient sediments.

Their evaluations were based on the following criteria:

1. context and geological robustness,
2. single domain (criterion SD),
3. size and shape (score S),
4. chain quality/length (score C),
5. chemical perfection (criterion ChP),
6. crystallographic perfection (criterion CrP).

Among these, the SD, S and C criteria are the most important as they are believed to offer the clearest evidence for a biological origin of magnetite or greigite crystals.

In order to test if these criteria can be validated also with experimental samples, where different environmental parameters have been varied during magnetite magnetosome growth, we have tested their validity by ascertaining whether MTB produced magnetites that had formed at different pH values (Moisescu et al., 2011) would pass the biogenicity tests based on the criteris set out by Kopp and Kirschvink (2008). The first criteria must naturally be ignored, because experimental MTB nanocrystals cannot be placed within a stratigraphic context and thus the paleomagnetic quality index cannot be attributed to the experimental samples, despite the fact that such an attribute is part of the first criterion.

However, when we considered the nanocrystals synthesized at optimal conditions (pH 7, xx mM Fe and *T* = 28°C) we found that with a S score of 4 and a C score of 3, anyone would judge the magnetite nanocrystals produced in experimental settings (Moisescu et al., 2011) to be magnetofossils. Such an evaluation would be valid regardless of which set of magnetofossil robustness criteria would be selected from Table 1.

**Table 1:** **Proposed magnetofossil robustness criteria (Kopp and Kirschvink, 2008)**.

**Context**	**Criteria**
Environment analogous to younger magnetofossil bearing environments;	^*^S ≥ 3; or
	S ≥ 2 and ^**^C ≥ 3; or
	S ≥ 2 and C ≥ 2 and ^***^ChP
Paleomagnetic data robust	
Environment analogous to younger magnetofossil bearing environments;	S ≥ 3 and ChP; or
	S ≥ 3 and C ≥ 3; or
	S ≥ 3 and C ≥ 2 and ChP
Paleomagnetic data not robust	
Environment analogous to younger magnetofossil-bearing environment;	S = 4 and ChP; or
	S ≥ 3 and C ≥ 3 and ChP; or
	S ≥ 3 and C ≥ 2 and ChP and ^****^CrP
Sediments have undergone burial metamorphism or paleomagnetic data remagnetized	
Unique environment	S = 4 and C ≥ 3 and ChP and CrP

* S, size and shape score;

** C, chain quality/length score;

*** ChP, chemical perfection criterion;

**** CrP, crystallographic perfection criterion.

Conversely, for the magnetosomes that we synthesized at optimal Fe concentrations and ambient temperatures but at a pH value of 6.0 (Moisescu et al., 2011), the TEM images confirmed that an important number of particles meet the requirements for single domain behavior, although the majority fell within the SPM. Thus, these magnetosome samples passed the SD test. Kopp and Kirschvink (2008) found that the size and shape of the magnetosomes was one of the most important criterions in evaluating biogenicity. Based on the narrowness of the size and shape distributions evidenced from the coercivity or ferromagnetic resonance (FMR) spectra, the presence of SD particles with truncated edges (e.g., cubo-octahedral or hexa-octahedral morphologies), elongated SD particles, and statistics for SD populations with narrow size and shape distributions they scored this criterion between 0 and 4 points. In our study (Moisescu et al., 2011) the magnetosomes formed at pH 6.0 revealed very few crystals with a cubo-octahedral like morphology or elongated shapes (the majority of the particles had irregular shapes) and the statistical analysis showed broad CSD and SFD. Thus these magnetites earned only 1 point for the S score. Similarly, score C (quality of chain identification) was graded from 0 to 4, with zero indicating the absence of chains, one is for either SEM or low-temperature thermal demagnetization indication of chains, two is for FMR or TEM indication of short chains of ambiguous origin, three is for FMR or TEM identification of short chains, and four is for TEM identification of long chains in magnetic extracts. TEM images of pH 6.0 magnetic extracts, showed particles in short chains of ambiguous origin, yielding a C score of 2. As no other mineral phase, except magnetite was detected in these samples, the pH 6.0 samples passed the ChP score. As to the crystallographic perfection, the majority of the crystals had many imperfections so they failed the CrP criterion. When tested against any of the sets of robustness criteria presented in Table 1 (after Kopp and Kirschvink, 2008), our pH 6.0 samples achieved a S score of 1 and a C score of 2. Thus, based on this criteria, these experimentally produced magnetite magnetosome samples could not be considered robust magnetofossils.

We performed a similar analysis for the magnette magnetosome samples that we produced at pH 8.0 and 9.0. Interestingly, only the pH 8.0 magnetites (with a S score of 2, and a C score of 2) would be judged as robust magnetofossils according to the first set of criteria (Kopp and Kirschvink, 2008). The pH 9.0 samples could be considered robust if only we give them a generous C score of 2 instead the more realistic score or of 1 (although only one or two very short and ambiguous chains could be identified in our TEM images), or a S score of 3 instead of 2 if we give points for the unique anisotropic prismatic crystals observed in these samples (Table 2, Figure 1D). Thus, such evaluations may not be the most adequate.

**Table 2:** **Magnetofossils scores of MTB magnetites that had formed in our cultures (Moisescu et al., 2011) at different pH values**.

**Sample**	**Stratigraphic context**	***SD***	**S**	**C**	**ChP**	**CrP**	**Age**	**Robustness**	**Source**
pH 7.0	ND	+	4	3	+	+	NA	Robust	Moisescu et al., 2011
pH 6.0	ND	+	1	2	+	−	NA	Not robust	Moisescu et al., 2011
pH 8.0	ND	+	2	2	+	−	NA	Not robust	Moisescu et al., 2011
pH 9.0	ND	+	2	1	+	−	NA	Not robust	Moisescu et al., 2011
Chalk deposits	^*^PQ 5	+	2	4	ND	ND	Cretaceous	Robust	Kopp and Kirschvink, 2008
Carbonate platform	ND	+	1	2	ND	ND	Paleoproterozoic	Not robust	Kopp and Kirschvink, 2008

For comparison, two examples of robust or not robust magnetofossil samples were included from the analyses of Kopp and Kirschvink (2008).

ND, not determined; NA, not applicable;

* paleomagnetic quality index.

Although all the samples from our work were clearly bacterial in origin, only two (i.e., pH 7.0 and 8.0) passed the magnetofossil robustness criteria evaluation, while the other two could easily be considered of abiotic origin. Consequently, not even these excellent criteria are always foolproof when it comes to precisely determine the biotic or abiotic origin of a magnetofossil. For that reason, we consider that a revision of these criteria, which should include more detailed analyses that should embrace other magnetosome qualities, is desperately needed if such criteria are to be used to identify biofossils.

## Biogeochemical cycling of Fe

For magnetosome synthesis, MTB require high quantities of Fe, which they usually rapidly convert to magnetite, a metabolic inert form that can no longer be used as an Fe source by other organisms (the “end of the road” for Fe; Martins et al., 2007). These observations suggest that MTB play an important role in the biogeochemical cycling of Fe in aquatic environments (Simmons and Edwards, 2006, 2007). Considering that the potential of MTB in the biogeochemical cycling of Fe could be quantitatively significant (e.g., 38 ± 28 μg Fe cm^−2^ year^−1^; Simmons and Edwards, 2007), an estimate of the MTB contribution to the Fe cycle became necessary.

In a previous study (Moisescu et al., 2011), using optimal MTB growth conditions we estimated that the MTB contribution to the biogeochemical Fe cycling was 1.6 μmol Fe L^−1^ year^−1^. This is equivalent to ~0.078 mg Fe L^−1^ year^−1^ Fe being tied up in an inert mineral form that becomes inaccessible for any further biogeochemical processes (Barbeau et al., 1996). This estimate could theoretically be used for calculations of the effect of the entire MTB population present in a natural environment that is characterized by a certain concentration of dissolved iron.

Nevertheless, in nature, pH, temperature, nutrients, oxygen levels or iron concentrations will vary due to short-time or seasonal environmental changes and thus any such estimate has to be carefully calibrated. We have therefore estimated in a first instance a potential pH-dependent contribution of MTB to the biogeochemical cycling of Fe and derived minimum and maximum values of 0.98 and 4.2 μmol Fe L^−1^ year^−1^ respectively. This corresponds to 0.06–0.24 mg Fe L^−1^ year^−1^ removed. A similar calculation, if we take only temperature fluctuations into account, leads to an estimate of the MTB contribution of between 0.3 and 4.2 μmol Fe L^−1^ year^−1^ (respectively 0.02–0.24 mg Fe L^−1^ year^−1^). All these estimates were based on values for Fe in aqueous media and thus they offer only a general view over the total annual iron cycling in aquatic environments and do not purport to present global perspective. However, despite being speculative, these estimates show the vast potential of MTB in sequestering Fe and thus in affecting and majorly impacting the global biogeochemical cycling of Fe.

## Summary and outlook

The first indication that bacteria may be capable of synthesizing intracellular minerals came with the discovery of MTB and was confirmed by the electron microscopy studies of Frankel et al. (1979), who provided the first proof of intracellular magnetite production in bacteria.

Magnetite and greigite magnetosomes from MTB have been optimized by evolution resulting in a perfect fusion of physicochemical and magnetic properties to support remarkable biological functions such as magnetotaxis. We discussed above how the intracellular metabolism and chemistry of MTB may be highly affected by the environmental conditions in which MTB cells are synthesizing their magnetic mineral particles and how these may end up having characteristics very different from those expected from a highly controlled BCM process. Taking all these aspects into account, the criteria to assess the origin of magnetites in environmental samples, need to be revised in order to derive a more accurate and reliable biogenicity indicator.

We are however, still only at the beginning of our journey to fully describe how these characteristics may vary depending on variations in external, environmental factors during magnetosome synthesis. We realize that more in depth studies are needed in order to describe in full details all possible variations and consequences of such variations on MTB related processes. In this way, will we be able to answer the question whether magnetites preserved in meteorites or in Earth's ancient rock record are biotic or abiotic in origin, and this might help us understand the origin of life on Earth or on other planets.

## Author contributions

Cristina Moisescu, Ioan I. Ardelean, and Liane G. Benning conceived and carried out the data analysis for a part of the experiments reviewed in this manuscript. Cristina Moisescu, Ioan I. Ardelean, and Liane G. Benning substantially contributed to the conception, drafting and critically revising the manuscript for it important intellectual content. Cristina Moisescu, Ioan I. Ardelean, and Liane G. Benning gave their approval of the final revised version of the manuscript to be submitted.

### Conflict of interest statement

The authors declare that the research was conducted in the absence of any commercial or financial relationships that could be construed as a potential conflict of interest.
